# Mutations in human lipoyltransferase gene *LIPT1* cause a Leigh disease with secondary deficiency for pyruvate and alpha-ketoglutarate dehydrogenase

**DOI:** 10.1186/1750-1172-8-192

**Published:** 2013-12-17

**Authors:** Yohan Soreze, Audrey Boutron, Florence Habarou, Christine Barnerias, Luc Nonnenmacher, Hélène Delpech, Asmaa Mamoune, Dominique Chrétien, Laurence Hubert, Christine Bole-Feysot, Patrick Nitschke, Isabelle Correia, Claude Sardet, Nathalie Boddaert, Yamina Hamel, Agnès Delahodde, Chris Ottolenghi, Pascale de Lonlay

**Affiliations:** 1 Reference Center of Inherited Metabolic Diseases, Imagine Institute, University Paris Descartes, Hospital Necker Enfants Malades, APHP, Paris, France; 2 Department of Biochemistry, Hospital Bicêtre, APHP, Le Kremlin Bicêtre, France; 3 Metabolic Biochemistry and INSERM U747, University Paris Descartes, Hospital Necker Enfants Malades, Paris, France; 4 Neurology Unit, Hospital Necker Enfants Malades, Paris, France; 5 Department of Molecular Genetics, CNRS, UMR 5535, Montpellier, France; 6 Imagine Institute, INSERM, 781, Paris, France; 7 Genomic Core Facility of the Imagine Institute, University Paris Descartes, Hospital Necker Enfants Malades, Paris, France; 8 Plateforme Bioinformatique Paris Descartes, Université Paris Descartes, Hospital Necker Enfants Malades, Paris, France; 9 Department of radiology, Imagine Institute, University Paris Descartes, Hospital Necker Enfants Malades, APHP, Paris, France; 10 Genetics and Microbiology Institute, Paris-Sud University, CNRS-UMR8621, Orsay, France

**Keywords:** LIPT1, Leigh disease, Pyruvate dehydrogenase, Alphaketoglutarate dehydrogenase lipoic acid

## Abstract

**Background:**

Synthesis and apoenzyme attachment of lipoic acid have emerged as a new complex metabolic pathway. Mutations in several genes involved in the lipoic acid *de novo* pathway have recently been described (*i.e., LIAS, NFU1, BOLA3, IBA57*), but no mutation was found so far in genes involved in the specific process of attachment of lipoic acid to apoenzymes pyruvate dehydrogenase (PDHc), α-ketoglutarate dehydrogenase (α-KGDHc) and branched chain α-keto acid dehydrogenase (BCKDHc) complexes.

**Methods:**

Exome capture was performed in a boy who developed Leigh disease following a gastroenteritis and had combined PDH and α-KGDH deficiency with a unique amino acid profile that partly ressembled E3 subunit (dihydrolipoamide dehydrogenase / DLD) deficiency. Functional studies on patient fibroblasts were performed. Lipoic acid administration was tested on the *LIPT1* ortholog *lip3* deletion strain yeast and on patient fibroblasts.

**Results:**

Exome sequencing identified two heterozygous mutations (c.875C > G and c.535A > G) in the *LIPT1* gene that encodes a mitochondrial lipoyltransferase which is thought to catalyze the attachment of lipoic acid on PDHc, α-KGDHc, and BCKDHc. Anti-lipoic acid antibodies revealed absent expression of PDH E2, BCKDH E2 and α-KGDH E2 subunits. Accordingly, the production of ^14^CO_2_ by patient fibroblasts after incubation with ^14C^glucose, ^14C^butyrate or ^14C^3OHbutyrate was very low compared to controls. cDNA transfection experiments on patient fibroblasts rescued PDH and α-KGDH activities and normalized the levels of pyruvate and 3OHbutyrate in cell supernatants. The yeast *lip3* deletion strain showed improved growth on ethanol medium after lipoic acid supplementation and incubation of the patient fibroblasts with lipoic acid decreased lactate level in cell supernatants.

**Conclusion:**

We report here a putative case of impaired free or H protein-derived lipoic acid attachment due to *LIPT1* mutations as a cause of PDH and α-KGDH deficiencies. Our study calls for renewed efforts to understand the mechanisms of pathology of lipoic acid-related defects and their heterogeneous biochemical expression, in order to devise efficient diagnostic procedures and possible therapies.

## Background

Pyruvate dehydrogenase complex (PDHc) deficiency most often occurs as an isolated enzyme defect caused by mutations in X-linked *PDHA1* [MIM 300502] and associates with a spectrum of clinical presentations ranging from fatal infantile lactic acidosis to mild psychomotor retardation and/or Leigh disease [1]. A smaller number of PDHc deficient individuals have mutations in *PDHB*, *PDHX*, dihydrolipoyl transacetylase (*DLAT*), dihydrolipoyl dehydrogenase (*DLD*), or *PDP1*, which is responsible for the reactivation of phosphorylated PDHc [1]. Lipoic acid is a sulphur-containing cofactor covalently attached to the PDHc E2 subunit (DLAT) and is functionally required. A defect of lipoic acid metabolism is expected to affect PDHc as well as the other key enzymatic complexes that have similar structure and are lipoic acid dependent, notably α-ketoglutarate dehydrogenase (α-KGDH), branched chain keto acid dehydrogenase (BCKDH) and the glycine cleavage system [2,3].

Two pathways lead to attachment of lipoic acid to apoenzymes in bacteria. In the endogenous or *de novo* pathway, biosynthesis of lipoic acid follows mitochondrial fatty acid synthesis (FASII) and iron sulphur cluster biosynthesis. The first lipoic acid specific enzyme of this pathway is a lipoyl(octanoyl)transferase which catalyzes the attachment of octanoate to specific lysyl residues in lipoate-dependent enzymes (bacterial LipB and likely LIPT2 in humans). The second enzyme involved is lipoic acid synthase (LipA in bacteria and LIAS in humans), which catalyzes the conversion of the octanoyl side chain to an active lipoyl. The enzyme is an iron sulphur protein with two [4Fe-4S] clusters [4]. The [4Fe-4S] cluster is a cofactor of LIAS as well as of many proteins involved in intermediary metabolism and oxidative phosphorylation, where it participates in electron transfer reactions and in the functions of complexes I, II and III [5]. Assembly of the [4Fe-4S] cluster involves a complex metabolic pathway that includes NFU1 (NFU Iron-Sulfur cluster scaffold homolog)*,* ISCU (Iron-Sulfur cluster scaffold homolog) and BOLA3 [3] (bolA homolog 3).

The exogenous or salvage pathway involves attachment of free lipoic acid to the specific lysine residues of the target proteins. It is still unclear to which degree the lipoic acid salvage pathway (*LplA* in bacteria) is conserved in humans, as the *LplA* orthologous gene, *LIPT1*, encodes a lipoyl transferase that uses free AMP-activated lipoic acid as a substrate, yet in yeasts, the *LIPT1* orthologue *lip3* may act downstream, rather than independently of the *de novo* pathway [6,7] (see Discussion). *LIPT1* is organized into four exons in humans, only one of which is coding, and maps to chromosome 2q11.2.

As yet, mutations have been described in genes involved in the *de novo* pathway, i.e., *NFU1, BOLA3, LIAS* and *IBA57*[2,3,8-11]*.* Here we describe a new lipoate-related disease that involves impaired lipoate attachment on PDHc and α-KGDH and is due to *LIPT1* mutations.

## Methods

This work has been approved by our institutional ethical committee after declaration to the Département de la Recherche Clinique et du Développement; informed consent was obtained from the parents.

### Biochemical analysis and respiratory chain investigation

PDH and αKGDH activities, E3 activity, polarographic and spectrophotometric assay of mitochondrial respiratory chain (MRC) complex activities were measured in leukocytes and skin fibroblasts according to standard procedures [12,13].

Lactate and pyruvate levels were determined in fibroblast supernatants by enzymatic methods.

Oxidation rates of butyrate (fatty acid), 3hydoxybutyrate (ketone body) and glucose, three main energetic substrates were measured in cultured patient-derived fibroblasts after incubation with 1 and 10 mmol/l 1-^14^C labeled substrates [1]. The ^13^C_6_-labeled leucine loading test was a modification of a previously described radioactive assay [14] with an isovaleryl-CoA derivative (3-hydroxyisovaleric acid) as the readout for BCKDH activity. We used stable isotope (^13^C_6_) labeled leucine as substrate (Eurisotop, Saint-Aubin, France), and measured the ratio between 3-hydroxyisovaleric acid stable isotope to natural ions after leucine loading by gas chromatography – mass spectrometry (300MS from Varian/Brüker Daltonics, Fremont, CA, USA).

### Molecular investigations

DNA was extracted from white blood cells, collected from the patient and her parents after informed consent. The known genes *PDHA1*, *PDHB*, *PDHX*, *DLAT*, *DLD*[1], *LIAS* (GenBank NG_032111.1), *BOLA3* (GenBank NG_031910) and *NFU1* (GenBank NG_031931.1) were directly sequenced using DNA extracted from white blood cells and intronic primers (purchased from Applied Biosystems, Forster City, CA).

Exome capture was performed with the SureSelect Human All Exon 50 Mb kit (Agilent Technologies) with SOLiD5500 (Life Technologies). 75 + 35 Paired-end reads were mapped on human genome reference (hg19 build) using LifeScope (Life Technologies). Filtering was performed with GATK tools and an in-house software (PolyWeb).

### Immunoblot analysis in LIPT1, NFU1 and PDHA1 mutant fibroblasts

Total protein extracts were prepared by lysing cultured fibroblasts generated from either control individual or from patients with either *LIPT1*, *NFU1*, or *PDHA1* mutations. Cell pellets were lysed in Triton X-100 lysis buffer (50 mM Tris-HCl, pH 7.4, 100 mM NaCl, 50 mM NaF, 5 mM EDTA, 40 mM glycerophosphate, 1 mM Na orthovanadate, 10^-4^ M PMSF, 10^-6^ M leupeptin, 10^-6^ M pepstatin A and 1% Triton X-100). 40 μg of total protein extracts were separated by SDS-polyacrylamide-gel electrophoresis and transferred to nitrocellulose membranes, blocked in TBS containing 5% nonfat milk for 1 hour at room temperature and incubated overnight at 4°C with primary antibodies. The following antibodies were used: anti-LIPT1 (Santa Cruz Biotechnology), anti-lipoic acid (Abcam) to identify the lipoate containing subunits of oxo acid dehydrogenases (PDHc, α-KGDHc and BCKDHc) in fibroblasts, anti-Pyruvate Dehydrogenase E1-α subunit (Abcam) and anti-α Tubulin (Sigma-Aldrich). Quantitative analyses of immunoblots were performed with the Odyssey infrared image system (LiCor) using DyLight™ 800 conjugated secondary antibodies from LiCor.

### Patient fibroblast: LIPT1 transfection and lipoic acid supplementation in the medium

Human fibroblasts from skin biopsies from a control individual and the patient were cultured in monolayer flasks with HamF10 medium containing 12% fetal calf serum and 100 UI/mL penicillin G and 100 μg/mL streptomycin. The flasks were incubated at 37°C with 5% carbon dioxide. Fibroblasts were transfected using pLenti-GIII-CMV-hLIPT1-HA vector (Applied Biological Material), and analysed after 36 h.

Control and patient fibroblasts were cultured with culture medium supplemented with lipoic acid (Sigma) to a final concentration of 10 and 100 μM. Biochemical investigations were analysed on cultured skin fibroblasts before and after three weeks of supplementation.

### Yeast model

Wild-type and *Δlip3* strains, deleted for *LIP3*, the gene orthologous to human *LIPT1*[15], were grown on glucose and ethanol medium for 3 and 7 days at 28°C and 36°C. Lipoic acid (2 ng/mL) was added in growth medium of *Δlip3* cells to test its potential therapeutic effect.

## Results

### Clinical phenotype

The patient, a boy, was the first child of non-consanguineous French parents. He was born at term after an uneventful pregnancy and spontaneous delivery with normal birth parameters. Psychomotor development was delayed and associated with hypotonia. At age 15 months, when the patient was referred to our unit, metabolic investigations were regarded as normal, including brain magnetic resonance imaging (MRI), muscle biopsy, plasma amino acids and urinary organic acids (data not shown). The course of the disease was marked by an episode of metabolic acidosis at 18 months of age that occurred in the context of vomiting and dehydration associated with gastro-enteritis. The patient had hyperlactatemia (8 mmol/L), liver cytolysis (AST 305 u/l, ALT 220 u/l, N < 40) and psychomotor regression that occurred suddenly and included severe trunk hypotonia, coma with dystonia and no head control. After a few days, he was bed- and wheel-chair bound, could not stand or sit unaided. Severe spastic tetraparesis and extrapyramidal syndrome were observed. He could not speak but understood simple orders. He was otherwise fully conscious, alert, and he could smile, laugh and follow with eyes. Major swallowing difficulties led to gastrostomy. Brain MRI revealed cerebellar atrophy, an important sus-tentorial cortical atrophy with ventricular dilatation, bilateral thalamic anomalies, bi-frontal white matter anomalies and delayed myelinisation (Additional file 1: Figure S1). During the decompensation, the MRS spectroscopy with long TE (144) showed a peak of lactate (Additional file 1: Figure S1).

### Biochemical parameters

The most striking biochemical features involved the plasma amino acid profile that associated increased levels of glutamine and proline at presentation as well as on repeated sampling, and low levels of lysine at presentation (Table 1). This profile was consistent with multiple α-keto acid dehydrogenase deficiency, particularly involving PDH and α-KGDH, thus suggestive of E3 subunit/DLD deficiency. However, no or only slight elevations were noted for glutamate, α-amino, α-hydroxy or α-ketoadipic acids, thus contrasting with the large increases and sometimes massive amounts observed in typical forms of NFU1 deficiency ([2] and data not shown). In addition, in sharp contrast to all previously reported patients with deficiency in the de *novo* lipoic acid synthesis pathway, in our patient glycine was not increased in CSF, plasma or urine (Table 1). This was not consistent with deficiency of the glycine cleavage system. Furthermore, the branched chain amino acids unexpectedly showed from very low to low-normal levels, thus not suggestive of BCKDH deficiency (Table 1).

**Table 1:** Relevant plasma, urinary and CSF amino acids and urinary organic acid levels

	**At presentation**		**Patient follow-up through age 4 years**	
	**(age 18 months)**			
***Plasma (μmol/L)***	**Patient**	**Reference interval**	**Patient**	**Patient**
			**Median**	**Range**
Glutamate	119	2 - 118	97.5	60 – 241
Glutamine	1228	334 - 666	820	615 – 1381
Proline	441	61 - 285	417.5	179 – 576
Glycine	144	149 - 301	263.5	144 – 356
Alanine	470	134 - 502	455	221 – 1086
Valine	64	158 - 310	128	32 – 155
Isoleucine	21	37 - 89	39.5	7 – 51
Leucine	45	68 - 168	67.5	15 – 76
Lysine	52	113 - 269	180.5	142 – 326
***CSF (μmol/L)***				
Glutamate	0	ND		
Glutamine	723	352 - 885		
Glycine	5	1 - 16		
Alanine	52	6 - 47		
***Urine (μmol/mmol of creatinine)***				
Glutamate	2	<11		
Glutamine	603	62 - 165		
Glycine	229	110 - 356		
Alanine	239	41 - 130		
α-ketoglutarate	6224	<79		
Llactate	252	<52		
Succinate	15	<97		
Fumarate	48	<7		

Reference intervals are provided as the range of the age-matched reference population. Median and range of plasma amino acid values are also provided for the patient samples (N = 8) collected during follow-up.

Mild metabolic acidosis (bicarbonates 16 mmol/L, N > 20 mmol/L) associated with increased level of lactate and pyruvate in blood (lactate 8 mmol/L, N < 2, pyruvate 0.44 mmol/L, N < 0.2), in urine (lactate 252 μmol/mmol of creatinine) and CSF (lactate 6.60 mmol/L, pyruvate 0.36 mmol/L). Urinary α-ketoglutarate was highly increased (Table 1). On subsequent tests, lactate and pyruvate levels as well as urinary α-ketoglutarate were normalized.

A severe decrease of PDH and α-KGDH activities was found in patient fibroblasts (Table 2), and BCKDH activity was also strongly reduced, as inferred by a ^13^C_6_ labeled leucine loading test that showed levels comparable to fibroblasts from a maple syrup urine disease patient. In contrast, mitochondrial respiratory chain activities and E3 subunit (DLD) activity measured in fibroblast homogenates were normal (data not shown). Polarographic assay in fibroblasts showed reduced oxygen production using pyruvate as substrate (2.2 nmol O_2_/min/mg of protein, reference range from 3.3 to 6.8 nmol O_2_/min/mg of protein).

**Table 2:** Biochemical investigations

	**Patient**	**Control**
**Enzyme activities (pmol/min/mg protein)**		
ketoglutarate dehydrogenase (α-KGDH)	900	7000
Isocitrate dehydrogenase (IDH)	21000	23000
Pyruvate dehydrogenase (PDH)	90	1117
Citrate Synthase (SC)	27000	48000
**Polarographic study (nmolO2/min/mg protein)**		
Pyruvate + malate	6.2	31.1 +/- 2
Malate + glutamate	5.4	28.9 +/- 4.5
**CO**_**2**_**production (nmol/h/mg of protein)**		
Butyrate	1.5	6
Octanoate	0.9	3.4
Palmitate	1.6	3.5
Glucose (1 mmol/l)	2.8	7.8
Glucose (10 mmol/l)	3.7	8.8
OH butyrate (1 mmol/l)	0.8	3
OH butyrate (10 mmol/l)	2.3	8.5

PDHc, α-KGDHc activities and polarographic studies on patient and control skin fibroblasts, CO_2_ production after administration of ^14^C-3OHbutyrate, ^14^C-glucose or ^14^C- butyrate in fibroblasts of the patient and a control.

**Figure 1:**
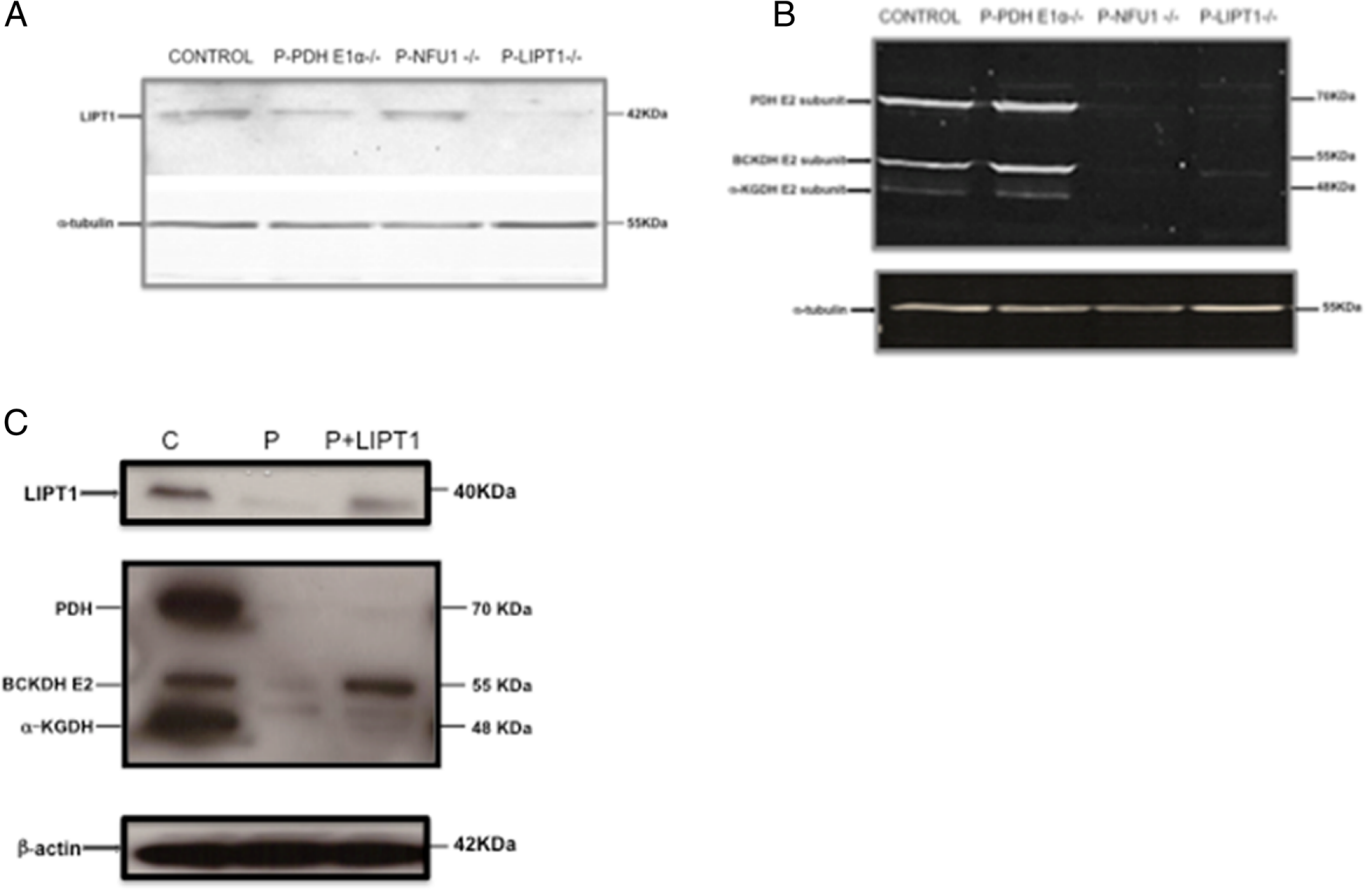
**Western blots of patient fibroblasts with antibodies against LIPT1 (A), lipoic acid (B), and tubulin (Sigma-Aldrich) or actine in cultures in basal conditions (A, B) and after *LIPT1 *transfection (C). A** and **B**: Control, patients (P) with *PDHA1*, *NFU1* and *LIPT1* mutations were as indicated. **A**: the LIPT1 antibody failed to detect the LIPT1 protein in patient fibroblasts. **B**: Anti-lipoate antibody failed to detect the expected lipoylated proteins of ketoacid dehydrogenases (PDHc, α-KGDHc and BCKDHc) in the patient fibroblasts as well as in fibroblasts of another patient with *NFU1* mutations, whereas normal bands were seen in patient fibroblasts with *PDHA1* mutations and in control. **C**: Control (C), Patient with *LIPT1* mutations (P), patient fibroblasts transfected with *LIPT1* (P + LIPT1). Anti-lipoate antibody revealed normalized and very moderately increased amounts of BCKDH (x8) and α-KGDH (x1.8) proteins, respectively, in patient fibroblasts after *LIPT1* transfection whereas no band was detected for PDH protein.

Oxidation rates of butyrate (fatty acid), 3-hydroxybutyrate (ketone body) and glucose were measured by incubating patient-derived fibroblasts with 1 and 10 mmol/l 1-^14^C labeled substrates. We found that CO_2_ production by the Krebs cycle and mitochondrial respiratory chain activity in fibroblast’s patient were very low compared to controls after administration of each of these substrates (Table 2), suggesting a defect in both the Krebs’ cycle and PDH.

### Molecular investigations

To identify the causative gene mutation, we first excluded the genes *PDHA1*, *PDHB*, *PDHX*, *DLAT*, *DLD*[1], *LIAS* (GenBank NG_032111.1), *BOLA3* (GenBank NG_031910) and *NFU1* (GenBank NG_031931.1) by direct sequencing using DNA extracted from white blood cells. Subsequently, exome capture was performed and resulted in a list of 25 candidate genes, including a mitochondrial protein, LIPT1 (MIM 610284). The compound heterozygous c.875C > G and c.535A > G transitions in *LIPT1* gene (NP_660200.1) on chromosome 2 resulted in a stop mutation (p.Ser292X) and the substitution of a threonine by an alanine (p.Thr179Ala) in the protein, respectively. Sanger sequencing confirmed these mutations in the affected individual and showed that the parents were heterozygous for either mutation. These changes were predicted to be “damaging” and “deleterious” by Polyphen and SIFT softwares, respectively, and were located in a highly conserved domain of the protein. In addition, these mutations were not found in 100 controls of French origin or in exome sequencing projects including at least 13 000 alleles [http://evs.gs.washington.edu/EVS/].

### Immunoblotting

A LIPT1 antibody failed to detect the LIPT1 protein in patient fibroblasts (Figure 1A). Anti-lipoate antibody failed to detect the expected lipoylated proteins of ketoacid dehydrogenases (PDHc, α-KGDHc and BCKDHc) in the patient fibroblasts as well as in fibroblasts of another patient with *NFU1* mutations, whereas normal bands were seen in patient fibroblasts with *PDHA1* mutations and in control (Figure 1B). By contrast anti PDH E1-α (Abcam) antibody detected normal bands in the *LIPT1*-mutated patient fibroblasts (data not shown).

### LIPT1 transfection

**Figure 2:**
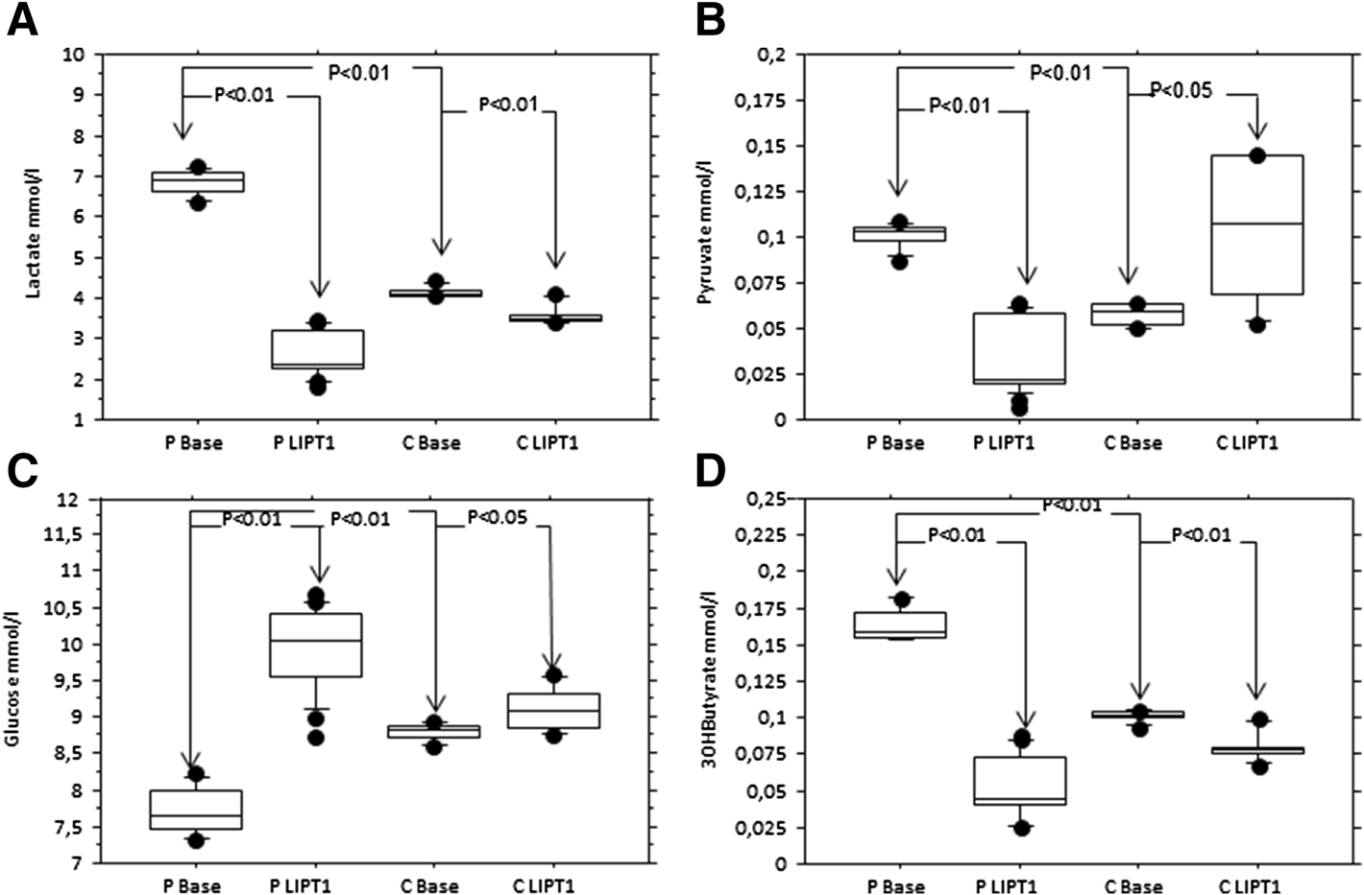
**Levels of lactate (A), pyruvate (B), glucose (C) and 3OHbutyrate (D) in supernatants from patient fibroblast’s cultured in basal condition and after *LIPT1 *transfection.** P: patient, C: control. **A**-**B**: Lactate (L) and pyruvate (P) levels were significantly increased in fibroblast supernatants compared to controls and they were dramatically decreased after *LIPT1* transfection. **C**-**D**: the level of 3OHbutyrate also decreased with a concomitant increase of glucose suggesting that the cells metabolized 3OHbutyrate upon partial rescue of α-KGDH activity. Data are represented as mean ± SEM.

Control and patient fibroblasts were transfected using the pLenti-GIII-CMV-hLIPT1-HA vector and analyzed after 36 h. As shown in Figure 1C after *LIPT1* transfection, anti-lipoate antibody revealed normalized (×8) and moderately (×1.8) increased amounts of BCKDH and α-KGDH proteins, respectively, in patient fibroblasts (quantification was performed with image J software) whereas no band was detected for PDH protein. PDH and α-KGDH activities increased moderately after *LIPT1* transfection (PDH: 90 and 93 to 210 and 215 pmoles/min/mg protein; α-KGDH: 0.9 to 3 nmoles/min/mg protein). Lactate (L) and pyruvate (P) levels were significantly increased in fibroblast supernatants compared to controls and they were dramatically decreased after *LIPT1* transfection (Figure 2A-B). Interestingly, the level of 3OHbutyrate also decreased with a concomitant increase of glucose suggesting that the cells metabolized 3OHbutyrate upon partial rescue of α-KGDH activity (Figure 2C-D).

### Lipoic acid administration on yeast model and human fibroblasts

**Figure 3:**
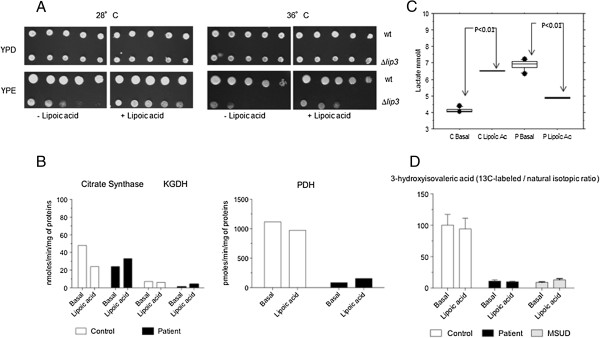
**Effects of lipoic acid administration on Δ*lip3-*deleted yeast strain and patient fibroblasts. A**: Growth of Δ*lip3* on glucose and ethanol at different temperature and after lipoic acid administration. Δ*lip3* yeasts failed to growth on ethanol at 28°C and the effect was stronger at 36°C. Lipoic acid was added in growth medium of Δ*lip3* strains and strikingly improved Δ*lip3* growth (2 ng/ml). **B**: PDH, α-KGDH and citrate synthase (CS) activities measured on cultured fibroblasts in basal condition and after administration of lipoic acid. In patient fibroblasts, this led to absent, or only moderate increase of α-KGDH and PDH activity. **C**: Lactate levels in cultured fibroblast supernatants in basal condition and after administration of lipoic acid. P: patient, C: control. Lactate level decreased significantly and this effect contrasted with the increase observed in control fibroblasts. Data are represented as mean ± SEM. **D**: Labeled to natural isotopic ratios for 3-hydroxyisovaleric acid (an isovaleryl-CoA derivative) in the *LIPT1*-deficient patient compared to a MSUD and control patient. The panel shows that *LIPT1* deficiency leads to a severe loss of metabolic flux involving BCKDH that is similar to *BCDKH* deficiency. The (+) and (-) signs stand for whether lipoate was added. Y-axis: ratio of stable isotope labeled vs natural 3-hydroxyvaleric acid. Error bars indicate standard deviations from triplicate experiments.

Wild-type and *Δlip3* strains, deleted for *lip3*, the gene orthologous to human *LIPT1*[15], were grown on glucose and ethanol medium for 3 and 7 days at 28°C and 36°C. A growth defect of *Δlip3* cells on ethanol was observed at 28°C and was exacerbated at 36°C (Figure 3A).

Lipoic acid (2 ng/mL) was added in growth medium of *Δlip3* cells to test its potential therapeutic effect and it markedly improved *Δlip3* growth (Figure 3A).

Because of this result, patient and control fibroblast cultures were supplemented with lipoic acid to a final concentration of 10 or 100 μM during three weeks. In the patient fibroblasts, this led to absent, or only moderate increase of αKGDH and PDH activities respectively (Figure 3B), yet lactate level decreased significantly and this effect contrasted with the lactate increase observed in control fibroblasts (Figure 3C). Nevertheless, the ^13^C_6_-labeled leucine loading test showed no effect of lipoic acid on BCKDH activity (Figure 3D). Thus, lipoic acid supplementation might have beneficial, though only partial effects, through mechanism(s) that remain to be determined.

## Discussion

We describe here a novel disease mechanism involving lipoic acid in humans that is induced by mutations involving the *LIPT1* gene. This increases the spectrum of the lipoic acid defects beyond those recently implicated in the *de novo* biosynthesis of lipoic acid, which include *LIAS* and the ancillary iron sulfur [Fe-S] cluster pathway (see above). We show that *LIPT1* mutations reduce lipoylation of PDHc and α-KGDHc, and result in severely decreased PDHc and α-KGDHc enzyme activities and abnormal pyruvate utilization by polarography.

**Figure 4:**
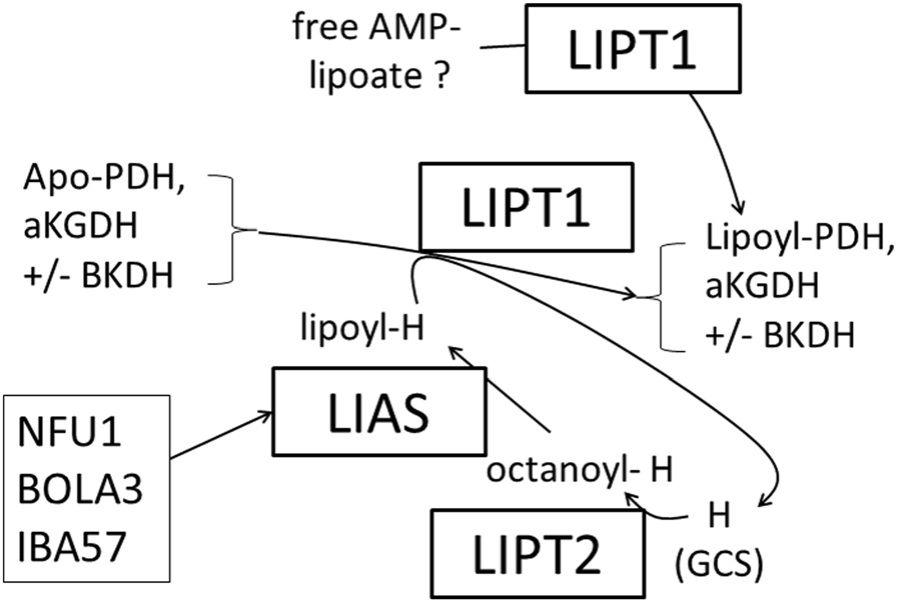
**Possible pathways for lipoic acid attachment to mitochondrial α-ketodehydrogenase apoenzymes.** AMP-activated forms of free lipoic acid could be a source of direct lipoylation via LIPT1 (the “lipoic acid salvage pathway”). This mechanism is at odds with some data in mice and yeast, yet it may account for apparent compensatory effects observed in the human disorders (see text). Another mechanism proposed in yeasts [15] may involve the H protein of the glycine cleavage system as a donor of lipoyl redidues to the other complexes via LIPT1. In this case, the *de novo* pathway involving LIPT2 and LIAS would act only on the H protein. This partly accounts for the different amino acid profiles observed in patients. The action of LIPT1 on BCKDH may be more complex than for other dehydrogenases (see text).

Based on available evidence [6,7], impaired attachment of non-protein bound, AMP-activated lipoic acid on mitochondrial proteins (thus affecting a putative lipoic acid “salvage” pathway) might be responsible for PDH and α-KGDH deficiencies in this patient. However, free lipoic acid does not seem to be able to compensate for other defects in the lipoylation pathway in yeast and mouse [5,11,15], whereas we show that it can partly rescue the growth of yeasts deficient for the *LIPT1* orthologue. Possible mechanisms are presented in Figure 4, which shows on the top, the putative salvage pathway of free AMP-activated lipoic acid, and on the bottom, an alternative or complementary mechanism with LIPT1 that redistributes lipoyl residues from the E2 subunit (H protein) of glycine cleavage system to the other complexes (as proposed in yeast [15]). In this mechanism, the original *de novo* pathway that involves LIPT2 and LIAS would act only on the H protein. Other mechanisms, such as partial redundancy between LIPT1 and LIPT2 operating either upstream of LIAS and/or in the usage of free AMP-lipoic acid as a substrate, cannot be excluded. None of these mechanisms accounts for the low branched chain amino acid levels in our patient and the greater susceptibility to BCKDHc lipoylation rescue of the patient fibroblasts (Table 1 and Figure 1C), suggesting that LIPT1 may be differentially required for BCKDHc *vs* α-KGDHc and PDHc (see also below).

The most striking biochemical features involved plasma amino acids that at presentation, associated increased levels of glutamine, proline with low levels of lysine and branched amino acids. Because of absent glycine increase, this profile more closely resembles the typical profile of E3 subunit/DLD deficiency rather than other genes involved in de novo biosynthesis of lipoic acid such as *NFU1*, *LIAS*, *IBA57*, *BOLA3* (as hyperglycinemia is a hallmark of the associated biochemical phenotype). Of note, the fact that elevated glycine (in several biological fluids) was the only observed amino acid abnormality in these diseases except for NFU1 deficiency, raises the possibility that when de novo lipoate synthesis is impaired, secondary deficiency of the dehydrogenase complexes other than the glycine cleavage system may be compensated for, though only partly, by *LIPT1-*mediated lipoylation. This is consistent with the possibility that part of LIPT1 action may be independent of *LIPT2* and *LIAS*, and this may be related to its ability to process free lipoate (Figure 4). In addition, the profile of our patient differed from E3 deficiency as branched chain amino acid levels were unexpectedly very low to low-normal, whereas they tend to be elevated in E3 deficiency, because of the associated BCKDH deficiency (which isolated, is responsible for maple syrup urine disease [16]). We verified that E3 activity was normal. Branched chain amino acid levels are known to be low in presence of BCKDH derepression following BKDK inactivation [17] and also in other conditions (e.g., nutritional deficiency), but none of these conditions can readily be reconciled with the proposed mechanisms of *LIPT1* deficiency (Figure 4), as well as with some of our in vitro studies (Figure 1C), or the overall amino acid profile shown by our patient at diagnosis and throughout follow-up (Table 1). Tissue specific differences may be involved, because basal BCKDH activity was clearly reduced in patient fibroblasts (Figure 3D).

Other explanations are possible and these results should be interpreted in the light that a large proportion of patients with related disorders such as E3 subunit (DLD) [18] and NFU1 deficiency have shown only transient or intermittent biochemical abnormalities (personal unpublished data), consistent with extensive heterogeneity of clinical and biochemical features in these diseases.

Based on our enzyme assays, CO_2_ production by the Krebs cycle and mitochondrial respiratory chain activity in patient fibroblasts were very low compared to controls after administration of each of three substrates (glucose, butyrate and 3OHbutyrate), suggesting a defect in both the Krebs’ cycle and PDH. Interestingly, the level of 3OHbutyrate also increased in fibroblast supernatant, presumably resulting from decreased α-KGDH activity. Furthermore, 3OHbutyrate level decreased with a concomitant increase of glucose in fibroblast supernatant after *LIPT1* transfection, consistent with the possibility that the cells metabolized 3OHbutyrate upon partial rescue of α-KGDH activity. The novel combination of biochemical tests presented here is a promising tool for Krebs cycle investigations, particularly for *in vitro* testing of potential therapeutic applications. From this test we have been able to infer that candidate dietary treatments for this metabolic disease, such as ketogenic diet, were likely detrimental. The argument was very low CO_2_ production after administration of ^C14^3OHbutyrate and ^C14^butyrate.

Contrary to our patient, who had biochemical abnormalities restricted to BCKDH, PDH and α-KGDH, the phenotype of the diseases that involve lipoic acid *de novo* biosynthesis show numerous additional dysfunctions that are related to the [Fe-S] cluster pathway. These additional dysfunctions [5,19] affect succinate dehydrogenase and aconitase, two proteins from the tricarboxylic acid cycle, as well as the respiratory chain complexes I-III [5,19]. This may explain why in these patients, neurological symptoms were associated with multisystem abnormalities [2,3,8,10,11]. By contrast the clinical picture of our patient was limited to brain thus similar to primary PDH deficiencies. The extreme severity of the neurological defects may result from the added contribution of α-KGDH deficiency which is expected to induce a stronger energetic defect compared to isolated PDH deficiency. Differential tissue-specificity of *LIPT1* function(s) *vs* the classical de novo lipoylation pathway might also be involved [20]. However it is of note that in our patient, biochemical clues expected from an energetic disease such as hyperlactatemia and hyperpyruvicemia, or urinary α-ketoglutarate were observed only during a metabolic attack. This intermittent and moderate biochemical presentation raises the possibility that the disease may be underdiagnosed. In fact, it was mainly the amino acid profile, partly suggestive of E3 subunit/DLD-like deficiency (see above), that prompted us to test for deficiency of α-KGDH and PDH.

A major challenge for this new and severe enzyme cofactor disease is therapy. A treatment with lipoic acid may not be indicated as its attachment to enzyme complexes is expected to be impaired in the absence of *LIPT1*. However *Δlip3* moderately improved growth in ethanol medium supplemented with lipoic acid, whereas activity of PDH, but not α-KGDH or BCKDH, moderately increased in patient fibroblasts cultured with lipoic acid supplementation in the medium. These preliminary results raise the possibility that lipoic acid may have therapeutic effects, though moderate and requiring further validation.

In conclusion, we report pathogenic *LIPT1* gene mutations in humans, which alter lipoate binding in PDHc and α-KGDHc, and we provide strong evidence for the existence of a lipoylation pathway specific to PDHc and α-KGDHc in humans. This pathway is at least partly distinct from the *de novo* pathway and does not affect lipoylation of the glycine cleavage complex, as was proposed in yeast [[15] and Figure 4]. In addition, our data indicate that *LIPT1* deficiency may affect lipoylation of BKCDHc in a partly unpredicted fashion that remains to be characterized. Further investigations are required to clarify the complex metabolism of lipoic acid cofactor in humans, and more efficient procedures should be devised for early diagnosis and prevention of metabolic decompensations.

## Abbreviations

PDH: Pyruvate dehydrogenase; αKGDH: α-ketoglutarate dehydrogenase; BCKDH: Branched chain α -keto acid dehydrogenases; DLD: Dihydrolipoamide dehydrogenase.

## Competing interests

All authors have no financial conflict of interest to disclose that might be construed to influence the results or interpretation of their manuscript.

## Authors’ contributions

YS, AM, LH, YH and PDL: exome analysis, PCR, cell culture, lipoic treatment, immunoblotting; PDL: identification of the mutations, conception of the work and draft of the manuscript; AB, FH, LN, DC, IC, CO: biochemical analysis; PDL, CO: draft of the manuscript; CB: clinician; CBF: exome sequencing; PN: informatics; HD, CS: immunoblotting; NB: radiology; AD: yeast study. All authors read and approved the final manuscript.

## Authors’ information

YS: Master student; AB (MD, PhD), FH (MD), LN (Ingenieur), DC (Ingenieur), IC (technician), CO (MD, PhD) are biochemists working in Necker or Bicêtre hospital and University; CB: neurologist, MD; CBF and PN: Plateforme Imagine Institute and Paris Descartes university; HD (Ingenieur), CS (PhD): researchers at Montpellier; AD (PhD): researcher at Orsay (yeast); NB (MD, PhD): radiologist. PDL (MD, PhD): chief of the reference center of metabolic diseases at Necker.

## Supplementary Material

Additional file 1: Figure S1 Brain MRI in the 33 months-old boy with *LIPT1* mutations reveals cerebellar atrophy, an important sus-tentorial cortical atrophy with ventricular dilatation, bilateral thalamic anomalies, bi-frontal white matter anomalies and delayed myelinisation on Sagittal T1 **(A)**, axial T2 **(B)**, and coronal Flair **(C)**. The MRS spectroscopy with long TE (144) performed at 17 months (during the decompensation) shows a peak of lactate **(D)**. Click here for file
