# Supplementary figures and images for: Mutations in human lipoyltransferase gene LIPT1 cause a Leigh disease with secondary deficiency for pyruvate and alpha-ketoglutarate dehydrogenase
Source: Orphanet J Rare Dis. 2013 Dec 17;8:192. doi: 10.1186/1750-1172-8-192 (PMC3905285; doi:10.1186/1750-1172-8-192)

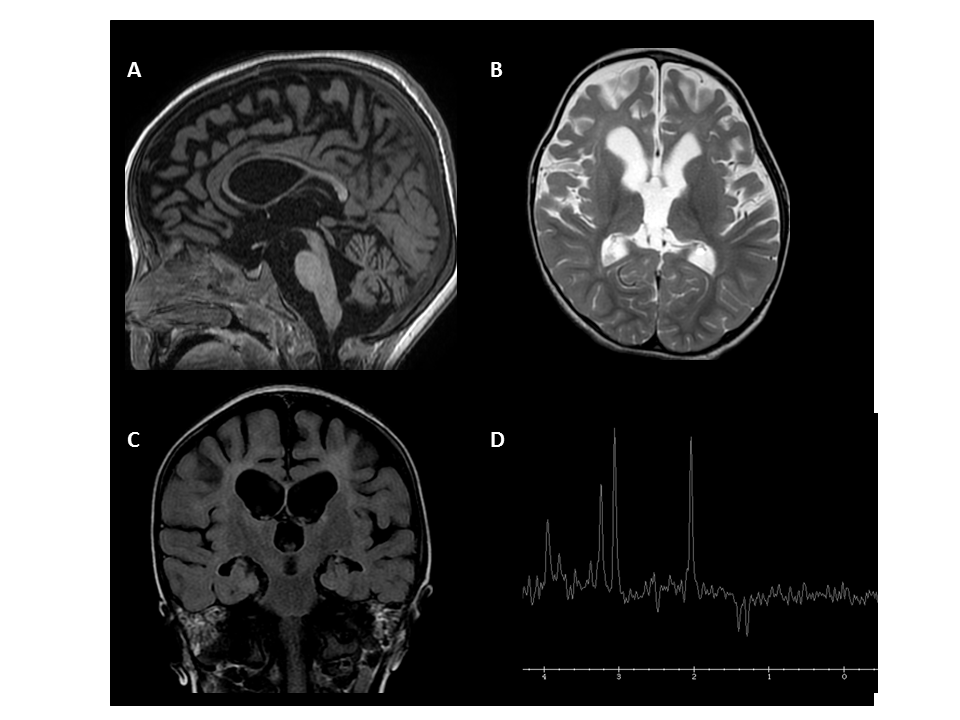

Supplement: Additional file 1: Figure S1 — Brain MRI in the 33 months-old boy with LIPT1 mutations reveals cerebellar atrophy, an important sus-tentorial cortical atrophy with ventricular dilatation, bilateral thalamic anomalies, bi-frontal white matter anomalies and delayed myelinisation on Sagittal T1 (A), axial T2 (B), and coronal Flair (C). The MRS spectroscopy with long TE (144) performed at 17 months (during the decompensation) shows a peak of lactate (D). [file 1750-1172-8-192-S1.tiff]
